# Submental liposuction for the management of lymphedema following head and neck cancer treatment: a randomized controlled trial

**DOI:** 10.1186/s40463-018-0263-1

**Published:** 2018-03-26

**Authors:** Uthman Alamoudi, Benjamin Taylor, Colin MacKay, Matthew H. Rigby, Robert Hart, Jonathan R. B. Trites, S. Mark Taylor

**Affiliations:** 1 Division of Otolaryngology – Head and Neck Surgery, Dalhousie University, Halifax, NS Canada; 2 Division of Otolaryngology – Head and Neck Surgery, Hail University, Hail, Kingdom of Saudi Arabia; 3 Division of Otolaryngology, Victoria General Hospital, Dalhousie University, Halifax, NS Canada

**Keywords:** Liposuction, Submental, Cervical, Lymphedema, Head and neck, Self-perception

## Abstract

**Background:**

Patients who have undergone treatment for head and neck cancer are at risk for neck lymphedema, which can severely affect quality of life. Liposuction has been used successfully in cancer patients who suffer from post-treatment limb lymphedema. The purpose of our study was to review the outcomes of head and neck cancer patients at our center who have undergone submental liposuction for post-treatment lymphedema and compare their subsequent results with a control group.

**Methods:**

All head and neck cancer patients at an oncology center in tertiary hospital setting who complained to their attending surgeon or radiation oncologist regarding cervical lymphedema secondary to head and neck cancer treatment, and had been disease-free for a minimum of one year, with no previous facial plastic surgical procedures were eligible for inclusion into the study. Study design was a non-blinded randomized controlled trial. Twenty patients were randomized into a treatment arm (underwent submental liposuction *n* = 10) and control arm (n = 10). Both groups of patients completed two surveys (Modified Blepharoplasty Outcome Evaluation and the validated Derriford Appearance Scale) on initial office visit after consenting for the trial. The treatment group then completed the surveys 6 months post-operatively while the control group filled the surveys 6 months after the initial assessment but had no intervention. Mann-Whitney U tests were performed to compare the responses of those that did and did not receive liposuction.

**Results:**

Our study demonstrated a statistically significant improvement in patients’ self-perception of appearance and statistically significant subjective scoring of appearance following submental liposuction.

**Conclusions:**

Submental liposuction is an effective and safe procedure to improves the quality of life for head and neck cancer patients suffering from post-treatment lymphedema.

## Background

According to Canadian Cancer Society data approximately 11,000 new cases of head and neck were diagnosed in Canada in 2015 [1]. The mainstay of treatment typically includes one, or a combination of, surgery, radiation, and chemotherapy. Each modality can lead to scar formation and affect lymphatic drainage pathways in the head and neck [2]. This can lead to internal (laryngeal or pharyngeal) or external (face and neck) lymphedema in up to 75% of patients [3]. Depending on the modality of the treatment, external lymphedema occurs in 6–54% of patients. Lymphedema in the face and neck may be disfiguring and often leads to negative self-perception of body image and social isolation. Secondary lymphedema affects patients’ physical and emotional well-being and has been shown to be associated with worse quality of life [2–9].

Many techniques have been used to assess and stage the lymphedema including radioactive lymphoscintigraphy, Indocyanine Green (ICG) lymphoscintigraphy, and MRI. These modalities are used to guide interventions, which include both non-invasive and reductive surgical approaches. One of the common reductive surgical procedures is liposuction.

Liposuction has been widely adopted for use in the upper extremity and more recently, the head and neck [4, 10–12]. A study by the senior author has shown submental liposuction to be efficacious in relieving emotional and physical stress associated with face and neck lymphedema among head and neck cancer patients [13]. The purpose of our study was to review the outcomes of head and neck cancer patients with post-treatment cervical lymphedema who were treated with submental liposuction compared to outcomes of similar patients who did not undergo liposuction therapy and therefore served as our control group. The decision to undertake such a trial was based on feedback from other surgeons who felt that the disease process may simply improve with time despite our belief that this is not the case.

## Methods

All head and neck cancer patients at our center who complained to their surgeon or radiation oncologist about cervical lymphedema following their head and neck cancer treatment, and had been disease-free for a minimum of one year, were eligible for inclusion into the study. Patients were excluded if they had undergone any previous or concurrent facial cosmetic procedures. With the approval of Nova Scotia Health Authority Research Ethics Board eligible patients were randomized using randomizer.org, accessed on May 16, 2013. Patients included in the study were asked to complete two validated surveys up to 3 time points during their regularly scheduled visits. The validated surveys included the Derriford Appearance Scale – DAS59 and the Modified Blepharoplasty Outcome Evaluation prior to the liposuction procedure, again at the time of surgery, and again six months or more following the procedure. The Derriford Appearance Scale is a validated scale designed to objectively measure psychological distress and dysfunction associated with aesthetic disfigurements and deformities [14]. It is subdivided into five categories: general self-consciousness; social self-consciousness; negative self-concept; sexual and bodily self-consciousness of appearance; and facial self-consciousness of appearance. It is intended and validated for persons over the age of 16. The scale for each question ranged from 1 to 5; a change of two between surveys was considered significant.

**Fig. 1:**
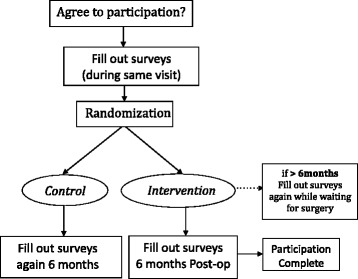
Methodology scheme

The Blepharoplasty Outcome Evaluation (MBOE) is a validated scale introduced by Ramsey Alsarraf in 2000 [15]. It was introduced as one of four facial plastic outcome measurement scales for the following procedures: rhinoplasty, blepharoplasty, facelift and skin rejuvenation. Each survey consisted of a similar six questions, each modified slightly as applicable for the various facial unit. It is a validated survey, which is used to monitor patients’ self-perception of appearance. The author was contacted and agreed to modification of the survey for the submental region with retained validity in our previous article [13]. The modified survey used in our study can be found in Table 1. Note: we did change the order of the scoring system of questions 3 and 5 that were counterintuitive to the general scoring of MBOE. Each question had a graded five-point response and a change of two points or more was considered significant. Refer to Fig. 1 for the scheme of our methodology.

All Participants completed questionnaires at the time of enrollment (Initial/Pre-treatment). Participants were then randomized to control and intervention (study) groups. For the intervention group, if the waiting time for the procedure was less than 6 months, then the participant underwent the liposuction procedure and was asked to complete the surveys 6 months following the procedure (Post-treatment). If the waiting time is longer than 6 months, participants were asked to complete the surveys (Awaiting-treatment) at the time of the procedure (Day of treatment), and at 6 months following the procedure (Post-treatment). Participants who were randomized to the control group completed questionnaires at the time of enrollment, and again 6 months later.

**Fig. 2:**
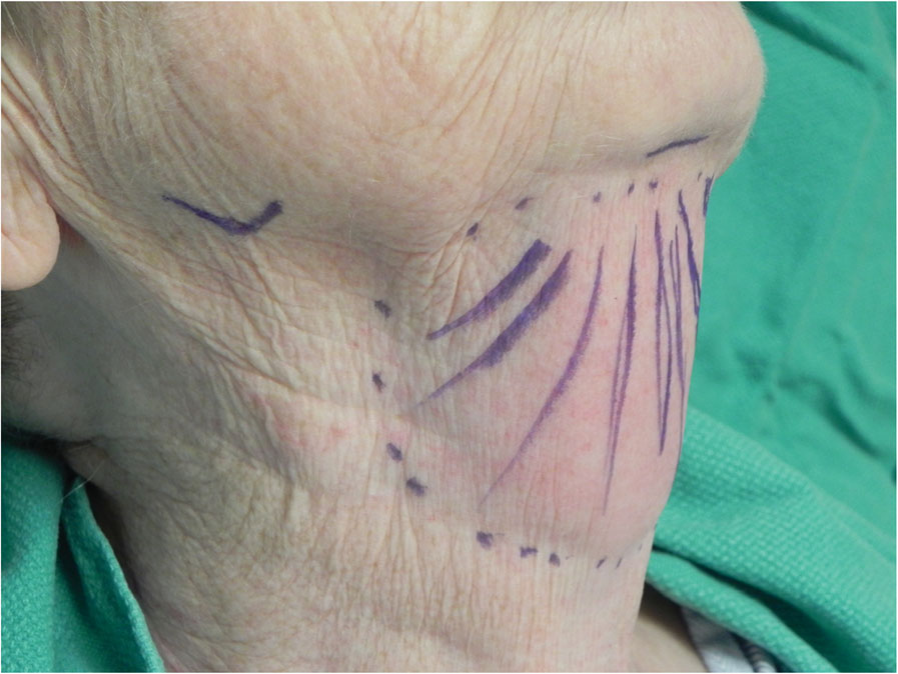
Surgical markings

**Fig. 3:**
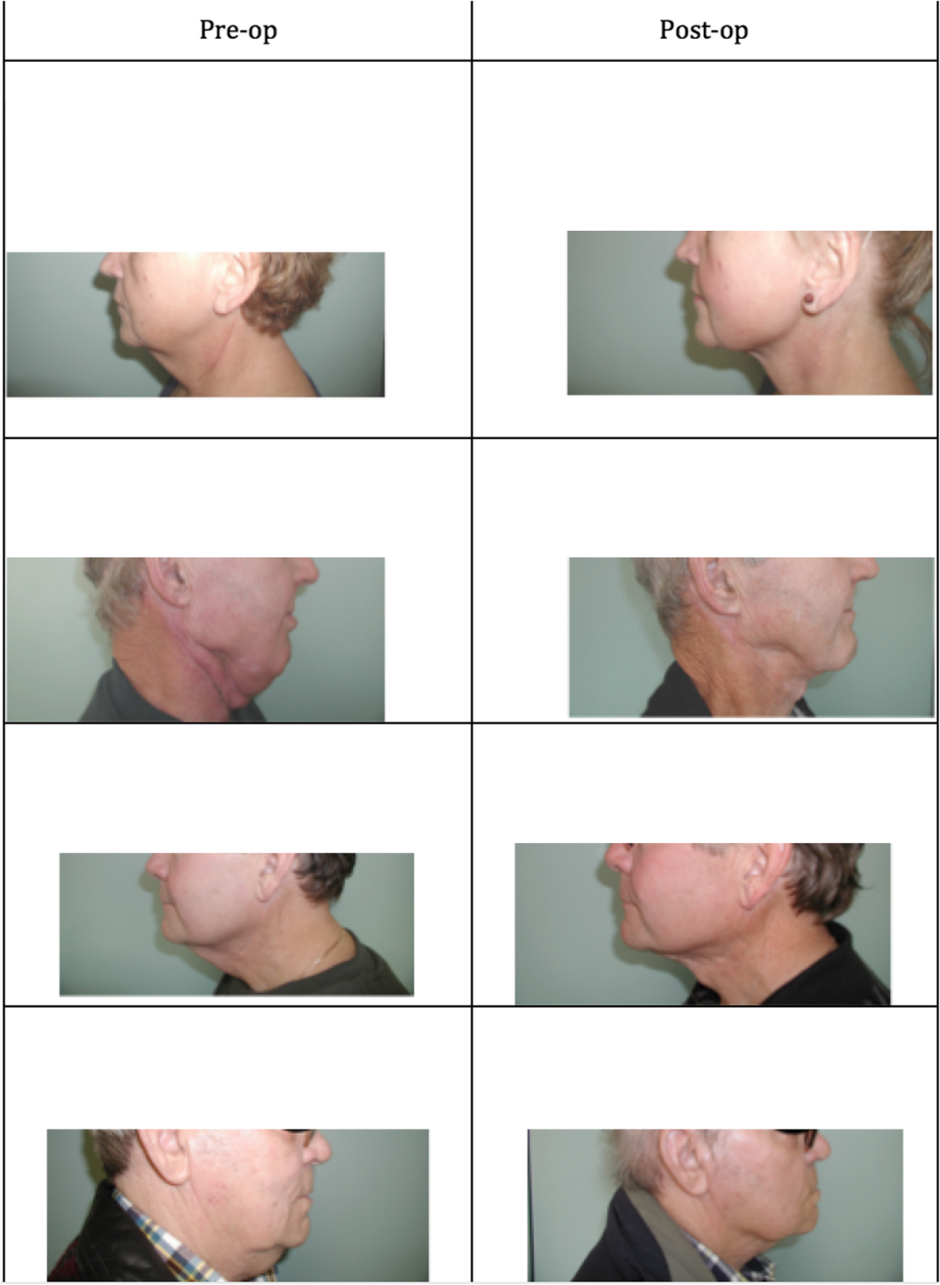
Photos showing the 4 patients prior to and after the liposuction procedure

Our surgical technique for submental liposuction for head and neck cancer patients has been previously described in the literature [4]. Submental liposuction is performed under local anesthesia as an outpatient operation. With the patient supine, a 5 mm incision was planned in a submental skin crease. The expected position of the marginal mandibular nerve was landmarked and marked bilaterally at the angle of the mandible (Fig. 2). After infiltration of local anesthetic, the skin incision is created within the submental skin crease. A 3 mm blunt-tipped Accelerator 3 (Mentor, Irving, Texas) liposuction cannula is introduced without suction. A fanning technique is used to break down adhesions and scarred tissue in the treatment area. The dissected area is then treated with suction applied to the cannula to remove fat. At the end of the case visual inspection is preformed to ensure a symmetric result. A facelift dressing is applied and is worn for one week post-operatively. Patients were prescribed a weeklong course of antibiotics (Fig. 3).

The objective of this study was to evaluate the outcomes of submental liposuction in post- treatment head and neck cancer patients with submental lymphedema, compared to control.

### Statistical analysis

Mann-Whitney U tests were performed to compare the responses of those that did and did not receive liposuction. For the MBOE survey, tests were performed for each of the five questions as well as the sum of all the questions. For the DAS-59 survey, tests were performed for each of the 59 questions, for the sum of each question in the 5 categories, and the overall sum of each question. For the demographics, a t-test was used to compare the mean age between study groups, and a Fisher’s exact test was used to compare the gender distribution and treatment distribution between study groups. IBM SPSS Statistics version 24.0 software (IBM Corp., Armonk, NY, USA) was used for the data analysis.

A power calculation was performed based on results from our pilot study. Using a two-tailed α of 0.05 and a β of 0.8, 10 patients divided equally between the control and experimental arms were required to detect a 1.3 difference in means of the MBOE scores the between arms.

## Results

Twenty-one patients were eligible for the study and all consented to participate. One of the participants died before completing the post-op evaluation and therefore was excluded from the study. The death was not related to the procedure described herein. Of the remaining twenty participants, seventeen were male and three were female. The high percentage of male participants in this study is most likely related to the preponderance of male patients treated for head and neck cancer at our institution. All patients in our group had undergone radiation therapy as a part of their cancer management; while eleven of the patients underwent both neck dissections and adjuvant radiation +/− chemotherapy. Their mean age was 64.9 (46–84) years. The time between completion of cancer treatment and liposuction therapy was 30 ± 12 months.

Primary sites included the oropharynx [16], larynx [6], neck [1], nasal cavity [1] and oral cavity [3]. The majority of patients were staged with T2 lesions [2]; four patients with T1, four patients with T3, and two with T4. One patient in the cohort had an unknown primary. Fifteen patients had nodal disease. None of the patients had distant metastatic disease.

There was no adverse outcomes encountered following submental liposuction. We assessed the difference in DAS-59 and MBOE scores of the two groups; those who had a 6 months waiting period without surgery (control group) and those who underwent liposuction (study group).

There were no significant statistical differences in distribution of examined demographic variables between the two study groups (Table 2).

**Table 1:** Modified blepharoplasty Outcomes Evaluation (MBOE)

1 How well do you like the appearance of your chin?					
Not at all 1	Somewhat 2	Moderately 3	Very 4	Extremely 5	N/A 0
2 How much do you feel your friends and loved ones like the appearance of your chin?					
Not at all 1	Somewhat 2	Moderately 3	Very 4	Extremely 5	N/A 0
3 Do you feel the current appearance of your chin limits your social or professional activities?					
Not at all 1	Somewhat 2	Moderately 3	Very 4	Extremely 5	N/A 0
4 How confident are you that the appearance of your chin is the best it can be?					
Not at all 5	Somewhat 4	Moderately 3	Very 2	Extremely 1	N/A 0
5 Would you like to surgically alter the appearance of your chin?					
Not at all 5	Somewhat 4	Moderately 3	Very 2	Extremely 1	N/A 0

For the MBOE scores, the overall summation of all five questions demonstrated a statistically significant improvement reported by patients that received liposuction compared to the control group (*p* < 0.001). Of the five individual MBOE questions, only question No. 3 (Do you feel the current appearance of your chin limits your social and professional activities) did not significantly improve the intervention group compared to the control group (*p* = 0.796) (Table 3).

**Table 2:** Participants demographic data

Total Participant	*N* = 20	*P*-value^$^
Age in years	64.9 (46–84)	0.072
Intervention	60.5 (46–77)	
Control	69.2 (58–84)	
Gender: M, F	17 (85%), 3 (15%)	0.211
Intervention	7 Male (70%)	
Control	10 Male (100%)	
Radiation +/_ Chemotherapy	9 (45%)	1.00
Intervention	5 (55.6%)	
Control	4 (44.4%)	
Radiation and Neck dissection	11 (55%)	
Intervention	5 (45.5%)	
Control	6 (54.5%)	
Stage of tumor	1 Tx 4 T3 4 T1 2 T4 9 T2	
Nodal disease	15	
	2 N1 7 N2b	
	2 N2a 4 N2c	

^$^T-test was used to compare the mean age and a Fisher’s exact test was used to compare the gender distribution and treatment distribution between two groups

For the DAS-59 scores, the overall summation of all the questions demonstrated a statistically significant improvement reported by patients that received liposuction when compared to the control group (*p* = 0.001). Summative scores in all the 5 categories of DAS-59 questions were significantly improved in the intervention group when compared to the control group(*p* < 0.05). Of the 59 individual questions, 21 showed statistically significant improvement in the intervention group when compared to the control group (Table 4). We want to emphasize that with this many tests performed in the DAS-59 analysis (65) that we would expect to observe that only 3 significant results would occur by chance alone.

**Table 3:** Mean differences in MBOE survey Responses

Modified Blepharoplasty Outcome Evaluation	Mean difference Liposuction group	95% CI	Mean difference Control group	95% CI	P-value^&^
1. How well do you like the appearance of your chin?	2.60	1.52–3.68	0.10	−.043–0.63	0.001
2. How much do you feel your friends and loved ones like the appearance of your chin?	2.20	1.09–3.31	−0.10	− 0.63–0.43	0.002
3. Do you feel the current appearance of your chin limits your social and professional activities?	0.20	−1.90 –2.30	0.10	−0.53–0.73	0.796
4. How confident are you that the appearance of your chin is the best that it can be?	2.60	1.70–3.50	−0.20	−0.65–0.25	0.000
5. Would you like to surgically alter the appearance of your chin?	2.70	1.44–3.96	−0.40	−1.37–0.57	0.002
Summation of the MBOE questionnaire	10.30	5.42–15.18	−0.50	−2.42 –1.42	0.000

^&^Individual response differences were analyzed using the Mann-Whitney U tests: Significant *P* values (.05 significance) are shown

*Abbreviations: CI: confidence interval. MBOE: Modified Blepharoplasty Outcome Evaluation*

**Table 4:** Mean differences in DAS-59 survey responses

DAS-59 Subsection	Intervention mean	95% CI	Control mean	95% CI	P-value^&^	Number of individual questions which reached statistical significance (*p* ≤ 0.05)
General self-consciousness appearance	−20.40	−30.83— -9.97	2.80	−5.86—11.46	0.001	11
Social self-consciousness of appearance	−9.30	−18.09— -0.51	3.40	−3.28—10.08	0.011	2
Sexual and bodily self-consciousness of appearance	−6.40	−9.38— -3.42	−0.40	− 4.86—4.06	0.011	3
Negative self-concept	−3.00	−5.00— -1.00	2.50	−0.72—5.72	0.007	4
Facial self-consciousness of appearance	−0.80	−2.58— 0.98	1.20	−0.34—2.74	0.043	1
Summation of all DAS-59 questions	− 41.00	− 62.92— -19.08	10.20	−8.28—28.68	0.001	21

^&^Individual response differences were analyzed using the Mann-Whitney U tests: Significant *P* values (.05 significance)

## Discussion

Lymphedema following treatment of head and neck cancer is a cause of disfigurement and anxiety to patients. The incidence of lymphedema depends on the modality of treatment required for management of the cancer and ranges from 6 to 54% [3–5]. Each modality can lead to disruption of lymphatic drainage pathways in the head and neck, and results in the collection of fluid and protein in the extravascular and interstitial spaces and subsequent lymphedema [2, 17].

Lymphedema has been shown to have negative physical and emotional effects. Lymphedema of the neck can lead to a negative self-perception and emotional distress. Physically, it can become severe enough to limit range of motion and function of the jaw, neck and shoulders. It can also cause otalgia, hearing loss, nasal congestion, and lead to dysphonia and dysphagia [4, 5].

Liposuction was introduced in 1988–1989 as an option for management of longstanding, stable, and incapacitating lymphedema of the arm following mastectomy [16] and also for lower extremity edema [10]. In a study by Brorson and Svensson, liposuction used in upper extremity lymphedema resulted in a reduction with liposuction and compression stockings compared to compression stockings alone. The results persisted up to 4 years on follow up. Additionally, they noticed a reduced incidence of cellulitis in patients who received liposuction therapy [11].

Submental liposuction in itself is not a new procedure. It has been used in cosmetic surgery for improvement of neck contour alone or in conjunction with other procedures. Patient selection in these cases is much more limited by patient age, skin tone, hyoid position or the amount of fat deposit on physical exam [13, 18]. In our patients, selection was not limited by the typical cosmetic criteria, and all were included if were medically fit, free of disease and interested in the procedure. As well, no other cosmetic procedures were performed on any of our patients to achieve the results. Our goal was to achieve acceptable functional and cosmetic results in the submental region as opposed to achieving the ideal anatomic contour following cosmetic facial plastic surgery. Pre-operatively, most of our patients had excessive tissue bulk, indurations and poorly defined normal landmarks and would be considered stage 3 on the Földi scale system.

One limitation of our study would be that we were not able to compare surgical intervention to non-invasive measures such as physiotherapy, which in our case is due to the unavailability of a dedicated physiotherapist who deals with lymphedema of the head and neck Additionally, we did not have blinded independent reviewers objectively score changes with and without the intervention being studied.

Submental liposuction has been shown to have fewer complications and is less invasive than traditional excisional techniques for lymphedema [4, 11, 13]. The documented complications include hematoma, scarring, cellulitis, necrotizing fasciitis, skin redundancy, platysmal banding and possible marginal mandibular nerve injury, [13, 18]. We did not have any complications in our study as a result of the liposuction procedure, and all our patients tolerated the procedure well under local anesthesia.

Similar to other studies that have been mentioned for other lymphedema sites, our submental liposuction procedures has successfully improved the quality of life and self perception of appearance in patients suffering from lymphedema secondary to head and neck cancer treatment.

Although we cannot comment on long-term results, this study shows much promise for improving the quality of life of head and neck cancer patients who are suffering for lymphedema. Liposuction showed statistically significant improvement in self-perception and self-confidence using the Modified Blepharoplasty Outcome Evaluation Scale and the validated Derriford Appearance Scale. We also hope to follow our currently enrolled patients to determine their long-term outcomes and benefits.

## Conclusions

Submental liposuction is a safe and effective procedure that appears to improve self-perception and self-confidence, which in turn improves the quality of life for head and neck cancer patients suffering from post-treatment lymphedema. We recommend this procedure for head and neck patients suffering emotionally or physically from post-treatment neck lymphedema.

## Abbreviations

DAS: Derriford Appearance Scale
MBOE: Modified Blepharoplasty Outcome Evaluation
